# Physical activity and left ventricular trabeculation in the UK Biobank community-based cohort study

**DOI:** 10.1136/heartjnl-2018-314155

**Published:** 2019-02-05

**Authors:** Simon P Woodbridge, Nay Aung, Jose M Paiva, Mihir M Sanghvi, Filip Zemrak, Kenneth Fung, Steffen E Petersen

**Affiliations:** William Harvey Research Institute, NIHR Barts Biomedical Research Centre, Queen Mary University of London, London, UK

**Keywords:** cardiac magnetic resonance (cmr) imaging

## Abstract

**Objective:**

Vigorous physical activity (PA) in highly trained athletes has been associated with heightened left ventricular (LV) trabeculation extent. It has therefore been hypothesised that LV trabeculation extent may participate in exercise-induced physiological cardiac remodelling. Our cross-sectional observational study aimed to ascertain whether there is a ‘dose–response’ relationship between PA and LV trabeculation extent and whether this could be identified at opposite PA extremes.

**Methods:**

In a cohort of 1030 individuals from the community-based UK Biobank study (male/female ratio: 0.84, mean age: 61 years), PA was measured via total metabolic equivalent of task (MET) min/week and 7-day average acceleration, and trabeculation extent via maximal non-compaction/compaction ratio (NC/C) in long-axis images of cardiovascular magnetic resonance studies. The relationship between PA and NC/C was assessed by multivariate regression (adjusting for potential confounders) as well as between demographic, anthropometric and LV phenotypic parameters and NC/C.

**Results:**

There was no significant linear relationship between PA and NC/C (full adjustment, total MET-min/week: ß=−0.0008, 95% CI −0.039 to –0.037, p=0.97; 7-day average acceleration: ß=−0.047, 95% CI −0.110 to –0.115, p=0.13, per IQR increment in PA), or between extreme PA quintiles (full adjustment, total MET-min/week: ß=−0.026, 95% CI −0.146 to –0.094, p=0.67; 7-day average acceleration: ß=−0.129, 95% CI −0.299 to –0.040, p=0.49), across all adjustment levels. A negative relationship was identified between left ventricular ejection fraction and NC/C, significantly modified by PA (ß difference=−0.006, p=0.03).

**Conclusions:**

In a community-based general population cohort, there was no relationship at, or between, extremes, between PA and NC/C, suggesting that at typical general population PA levels, trabeculation extent is not influenced by PA changes.

## Introduction

The degree of left ventricular (LV) trabeculation varies among individuals. The most documented pathological consequence of excessive trabeculation as a diagnostic phenotype is left ventricular non-compaction (LVNC), recognised as a genetic cardiomyopathy by the American Heart Association 1 and an unclassified cardiomyopathy by both the WHO and the European Society of Cardiology.^2 3^ Prominent trabeculation is sometimes observed in the setting of dilated and hypertrophic cardiomyopathies and in association with congenital heart defects and neuromuscular disorders.^4^ Therefore, it is plausible that trabeculation extent may be a morphological expression of a wide spectrum of myocardial disease.

More recently, there is growing interest in the role of physiological influence on cardiac trabeculation. It has been shown that postnatal changes in cardiac loading conditions affect trabeculation extent using models of pregnancy and extreme physical activity (PA).^5 6^ Previous literature has only identified a link between PA and trabeculation extent at the extreme level, using a cohort of highly trained athletes. To date, there has been no focused investigation into this relationship within a community-based population cohort, with PA distribution more reflective of a general population. For this study, such a cohort was provided by the UK Biobank study, a large prospective population-based cohort study of over 500 000 participants aged 40–69 years who have been recruited from 2006 to 2010. It aims to follow the health of these participants through comprehensive data collection at 22 centres across the UK, the core of which includes a wealth of demographic, anthropometric and environmental exposure data. A subset of these participants underwent cardiac magnetic resonance (CMR) scanning, which provided additional extensive cardiac phenotypic data.^7^

Our study thus aimed to determine for the first time, in a community-based population cohort, whether the amount of PA undertaken by an individual resembled a relationship of a ‘dose–response’ nature, as well as at high and low extremes of PA, with the degree of LV trabeculation extent observed, quantified by high-resolution (CMR) imaging.

## Materials and methods

### Participant selection

The UK Biobank has collected a wealth of phenotypic and genotypic information about its population of over 500 000 enrolled individuals, including data collection from questionnaires, physical measures, accelerometry, imaging, genome-wide genotyping with subsequent longitudinal follow-up for health-related outcomes. The sample size of 500 000 was theoretically calculated for reliable detection of the effects of different exposures on a wide variety of conditions in nested case–control studies with sufficient statistical power. The cohort’s characteristics make it well-suited to study exposure–disease relationships due to its large size and heterogeneity of exposure measures. The baseline summary characteristics of the cohort can be viewed on the UK Biobank website, in the data showcase section.^8^ The CMR imaging substudy of 5065 participants occurred between 2014 and 2015. The study complies with the Declaration of Helsinki and was approved by our institutional review body, with all participants having provided informed written consent.

### Classifying PA level

For each individual, PA was measured subjectively by both total metabolic equivalent of task (MET) min per week using information gathered from a self-reported International Physical Activity Questionnaire^9^ and objectively by 7-day average acceleration in units of milligravity measured by a wrist-worn triaxial accelerometer. The MET is a physiological measure of energy expenditure assigned to a particular PA, which compares its relative intensity compared with rest; this was combined with the duration of PA undertaken in minutes to form the composite measure of MET-min. This provides a generalised measure of activity volume undertaken, for instance, 30 min of brisk walking per day would equal 1050 MET min/week, and jogging for the same period every day would equal 1470 MET min/week. Table 1 and both online figures 1 and 2 in the supplementary material outline the calculation process.

10.1136/heartjnl-2018-314155.supp1 Supplementary data

The 7-day average acceleration measurement was acquired by an Axivity AX3 accelerometer worn continuously on the wrist of the participant’s dominant hand for seven consecutive days, detecting movement in all three axes. The raw data obtained from the device captured at 100 Hz was calibrated to adjust for acceleration due to local gravity using the van Hees method.^10^ Wear and non-wear episodes were also identified from the data.^11^

### CMR and trabeculation extent analysis

For all participants, all CMR studies were acquired with a wide bore 1.5 Tesla scanner (MAGNETOM Aera, Syngo Platform VD13A, Siemens Healthcare, Erlangen, Germany) and the postprocessing software cvi^42^ (version 5.1.1, Circle Cardiovascular Imaging, Calgary, Canada) was used for scan analysis. LV mass, LV end-diastolic volume (LVEDV) and LV ejection fraction (LVEF) were manually measured from balanced steady-state free precession cine short-axis and long-axis images.

**Figure 1:**
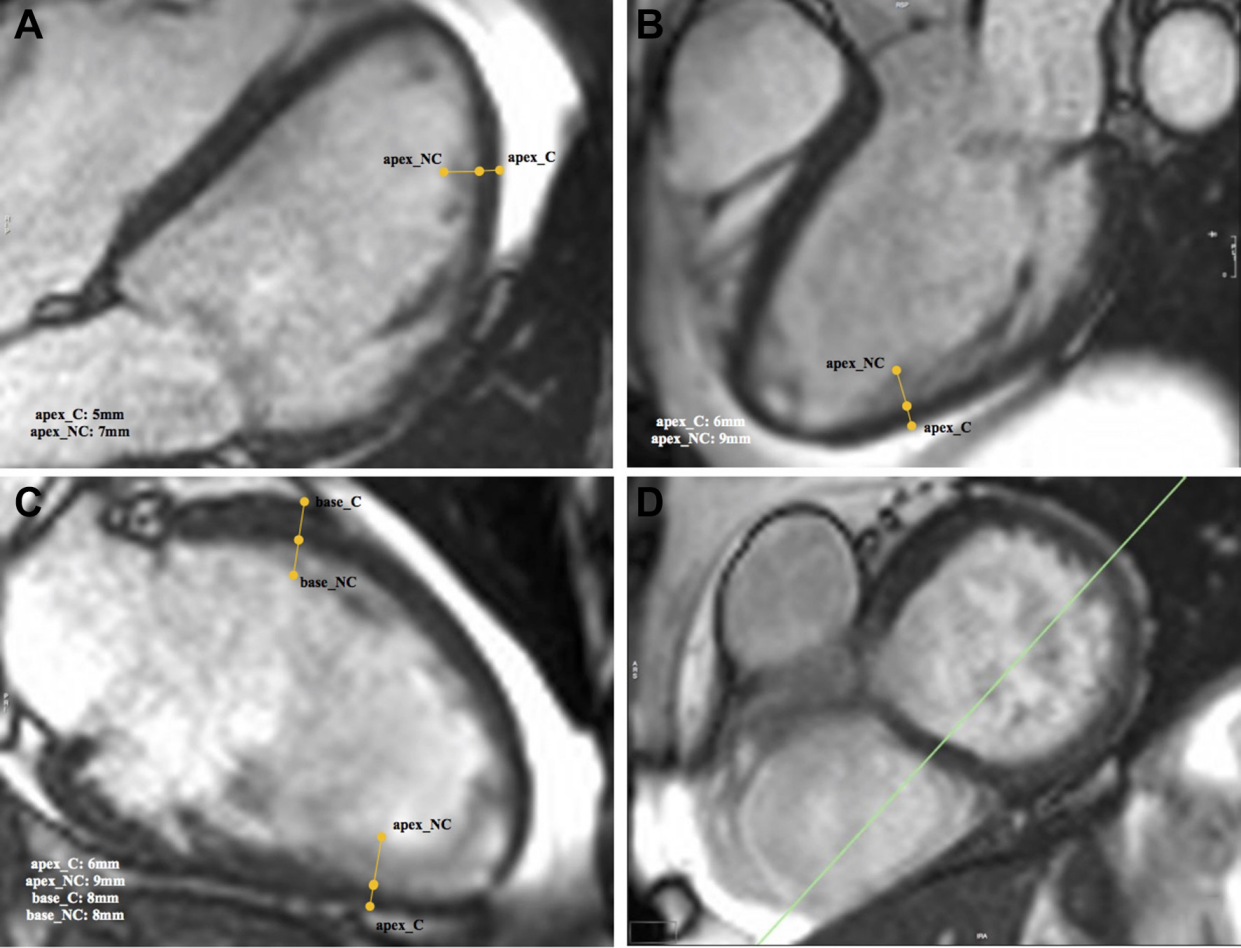
A demonstration of the measurement of the widths of the non-compacted and compacted layers using the cvi^42^ software. Measurements are indicated by the yellow lines. Measurements were labelled in the format ‘(region)_(compaction level)’; for example, ‘apex_NC’ indicates a non-compacted layer in the apex. Figure parts A–C indicate the 4, 3 and 2 chamber long-axis views, respectively, and (D) indicates the short-axis view for reference. The widths of each measurement are displayed at the bottom-centre of each long-axis view. These were used to calculate the NC/C ratio. NC/C, non-compaction/compaction.

These long-axis images were also used to measure trabeculation extent, using the maximal non-compaction/compaction (NC/C) ratio. There was an initial qualitative assessment for the presence of trabeculation in each of the three LV regions (basal, mid and apical) in the end-diastolic phase, defined as the visual identification of two myocardial layers represented by a difference in signal intensity between the two layers. If a trabeculated layer was visualised, the point at which the highest NC/C ratio could be obtained was measured for each region.^12^ The NC/C ratio was obtained by measuring the widths of the non-compacted (trabeculated) and compacted layer using the line contour tool, perpendicular to the length of the compacted layer. The highest ratio from any region of the whole scan was used to represent the trabeculation extent for that scan in the statistical analysis. Figure 1 demonstrates the process.

### Statistical analysis

All continuous variables were assessed for normality using histograms and quantile–quantile plots. Descriptive statistics for continuous variables were presented as mean±SD or median±IQR, while categorical variables were presented as a frequency (percentage). Missing data, if encountered, was addressed using multiple imputation by chained equation technique to create statistically valid imputed data based on variables from the original observed dataset to form 20 differently imputed datasets. The estimates and SEs from each imputed dataset were then pooled with Rubin’s rules.^13^

We constructed multiple nested linear regression models to identify any potential ‘dose–response’ relationship between the NC/C ratio and each PA measurement variable. We scaled both PA variables by their IQR before entering into the models to enable sensible comparison of effect estimates between the two methods of PA measurement; the effect estimates therefore indicate the change in relevant dependent variable per IQR increment in PA. In the unadjusted models, the bivariate association between PA and maximal NC/C ratio was tested. In limited models, adjustments were made for demographics and anthropometrics: age, sex, ethnicity and height. In fully adjusted models, the remaining covariates were added: body mass index (BMI), systolic blood pressure, diastolic blood pressure, heart rate (HR), smoking status, regular alcohol use, degree level education, diabetes, cardiovascular disease, hypertension, income, Townsend deprivation index, antihypertensive use, statin use, LVEDV, LV mass and LVEF.

We performed the following secondary analyses: (1) an investigation of the association between NC/C ratio and high and low PA quintiles (for each PA measurement method) using multivariate linear regression, following the same levels of adjustment, to form the extreme groups analysis, (2) an analysis of effect modification by age, sex, BMI, LVEDV, LV mass and LVEF by introducing cross-product terms to the dose–response linear regression analysis, (3) an investigation of the fully adjusted models of multivariate regression analysis between PA and maximal NC/C ratio to observe any significant associations between the outcome and the included covariates, (4) an investigation of the relationship between PA and clinical cardiac parameters (LVEDV, LV mass and LVEF) to clarify whether typical physiological changes that occur in exercise-induced cardiac remodelling were exhibited in our cohort and (5) an investigation of ‘dose–response’ relationships (using the same method of model construction) between PA in MET-min and maximal NC/C ratio at walking, moderate and vigorous PA intensities and between categorical predefined PA intensity levels and maximal NC/C ratio (of which the methodology is described in online appendix 1 of the supplementary material).^14^ We performed restricted cubic spline transformation of PA variables to investigate non-linear relationships. The optimal number of knots for restricted cubic spline-transformed variables was determined by the Akaike information criterion. Twenty randomly selected studies were independently analysed by two different readers to assess the reproducibility of NC/C ratio measurements. Interobserver variability of repeated measurements was quantified by intraclass correlation coefficient (ICC) and was visually assessed with a Bland-Altman plot. The programming language R was used for all statistical analyses.^15^ P values below 0.05 were considered statistically significant. We estimated that a sample size of at least 1000 would provide 99.8% statistical power at small effect sizes (R^2^=0.05). Online table 2 in the supplementary material demonstrates the output of power calculations based on a range of R^2^ values and effect sizes.

## Results

A total of 1030 participants were selected randomly for analysis in this study, from the initial pool of 5065 UK Biobank participants who had undergone CMR imaging. Online figure 3 in the supplementary material summarises the selection process.

### Baseline characteristics

Table 1 shows the baseline characteristics summary for both the whole dataset and a subset of this dataset, which includes participants for which there was no missing data in all covariate fields, termed ‘complete cases’. Both datasets showed a statistically significant difference—but minimal clinical difference—between the mean ages only, while means for LVEDV, LV mass, LVEF, maximal NC/C ratio, total MET-min/week and overall acceleration average did not differ significantly between datasets. The original cohort was predominantly of Caucasian ethnicity, with a mean age of 61 years, where 45.7% were men.

**Table 1:** Baseline characteristics for whole dataset and complete cases (of exclusively no missing covariate data)

	Whole dataset	Complete cases	P value	Percentage complete (%)
n	1030	437		
Age, years (mean [SD])	61 (7.6)	59 (6.4)	<0.001	100
Male sex (n [%])	471 (45.7)	191 (43.7)	0.513	100
Caucasian ethnicity (n [%])	1023 (99.3)	430 (98.4)	0.171	100
Height, cm (mean [SD])	169.8 (9.3)	169.8 (9.1)	0.950	100
BMI, kg/m^2^ (mean [SD])	26.7 (4.2)	26.4 (4.0)	0.274	100
Weight (mean [SD])	75.5 (14.7)	74.8 (14.3)	0.386	100
Systolic blood pressure, mm Hg (mean [SD])	136.4 (18.0)	135.2 (17.7)	0.250	99.9
Diastolic blood pressure, mm Hg (mean [SD])	78.7 (9.7)	78.7 (9.3)	0.990	99.9
Heart rate, bpm (mean [SD])	70 (11.8)	70 (11.4)	0.902	100
Average household income before tax (n [%])			0.726	89.3
Less than £18 000	129 (14.0)	56 (12.8)		
£18 000 to £30 999	270 (29.3)	117 (26.8)		
£31 000 to £51 999	285 (31.0)	139 (31.8)		
£52 000 to £100 000	180 (19.6)	96 (22.0)		
Greater than £100 000	56 (6.1)	29 (6.6)		
Degree level or professional education (n [%])	652 (63.3)	290 (66.4)	0.290	100
Townsend deprivation index (mean [SD])	−1.87 (2.68)	−1.76 (2.60)	0.472	100
Smoking status (n [%])			0.731	98.4
Never	611 (60.3)	255 (58.4)		
Previous	361 (35.6)	161 (36.8)		
Current	42 (4.1)	21 (4.8)		
Regular alcohol use (n [%])	446 (43.8)	189 (43.2)	0.888	98.8
Diabetes mellitus (n [%])	56 (5.4)	23 (5.3)	0.993	100
Cardiovascular disease (n [%])	91 (8.8)	24 (5.5)	0.038	100
Hypertension (n [%])	322 (31.3)	135 (30.9)	0.938	100
Antihypertensive use (n [%])	237 (23.0)	97 (22.2)	0.786	100
Statin use (n [%])	225 (21.8)	77 (17.6)	0.078	100
LVEDV, mL/m^2^ (mean (SD))	145.2 (33.4)	145.9 (33.0)	0.707	98.2
LV mass, g/m^2^ (mean [SD])	89.2 (24.1)	88.5 (23.4)	0.595	98.2
LVEF, % (mean [SD])	59.4 (5.9)	59.5 (5.6)	0.968	98.2
Maximal NC/C ratio (mean [SD])	1.93 (0.50)	1.91 (0.51)	0.508	100
Total MET-min/week, MET-min/week (median [IQR])	2115 (3599)	2226 (4061)	0.214	73.1
Seven-day average acceleration, milligravity (median [IQR])	27.56 (13.50)	28.41 (14.40)	0.164	62.6

‘Percentage complete’ refers to the proportion of complete data in our whole dataset per covariate.

BMI, body mass index; LV, left ventricular; LVEDV, LV end-diastolic volume; LVEF, LV ejection fraction; NC/C, non-compaction/compaction.

Splitting the range of values for both PA measurement methods produced the following quintiles: for total MET-min/week, these were 0–794.4, 794.4–1626, 1626–2853, 2853–5193 and 5193–24318 MET-min/week, and for average acceleration, these were 0.05–20.7, 20.7–25.7, 25.7–30.6, 30.6–37.6 and 37.6–67.1 units of milligravity. Online figure 4 in the supplementary material demonstrates the similar distribution of PA in total MET-min/week across the cohort, relative to the original CMR pilot study cohort of 5065 individuals. Maximal NC/C values ranged from 0.71 to 3.67, with a median of 2.00. There was a high level of agreement between repeated measurements of NC/C values as indicated by ICC (0.75) and the Bland-Altman plot in online figure 5 in the supplementary material.

### ‘Dose–response’ and extreme groups relationship analysis

Pooled multivariate regression was carried out between total MET and 7-day average acceleration, and maximal NC/C ratio, after multiple imputation. For both methods of PA measurement, there was no significant linear relationship demonstrated between PA and maximal NC/C ratio at all adjustment levels. Additionally, restricted cubic spline analyses showed no convincing evidence to support non-linear relationships (online figure 6 in the supplementary material).

Pooled multivariate regression was also performed between the lowest and highest PA quintiles of the cohort and maximal NC/C ratio for both PA measurement methods after multiple imputation. At all levels of model adjustment for both methods of PA measurement, no significant relationship was found between PA and maximal NC/C ratio between lowest and highest quintiles of PA values. Table 2 shows the analysis of both the dose–response and the extreme groups relationship.

**Table 2:** Association between PA (measured in both total MET-min/week and overall 7-day average acceleration) and maximal NC/C ratio for both the dose–response and extreme groups analysis

Clinical cardiac parameter	PA measurement method	Model								
		Unadjusted			Limited adjustment			Full adjustment		
		Effect Estimate	95% CI	P value	Effect Estimate	95% CI	P value	Effect Estimate	95% CI	P value
Dose–response analysis	Total MET-min/week	0.019	−0.019 to 0.056	0.32	0.024	−0.013 to 0.060	0.20	−0.001	−0.039 to 0.037	0.97
	Seven-day average acceleration	−0.012	−0.062 to 0.039	0.65	−0.007	−0.059 to 0.045	0.79	−0.047	−0.110 to 0.015	0.13
Extreme groups analysis	Total MET-min/week	0.049	−0.060 to 0.158	0.38	0.062	−0.046 to 0.171	0.26	−0.026	−0.146 to 0.094	0.67
	Seven-day average acceleration	−0.036	−0.157 to 0.086	0.56	−0.017	−0.144 to 0.111	0.79	−0.129	−0.299 to 0.040	0.49

Effect estimate reflects an NC/C ratio change per IQR of either total MET-min/week or 7-day average acceleration.

MET, metabolic equivalent of task; NC/C, non-compaction/compaction; PA, physical activity.

### Effect modification by cross-products

In fully adjusted models, age, sex, BMI, LVEDV and LV mass all produced no significant effect modifications on the continuous relationship between PA (in both measurement methods) and maximal NC/C ratio. LVEF was the only covariate to produce a significant effect modification when PA was measured in total MET-min/week. There was an overall negative relationship between LVEF and NC/C ratio, where one SD increment in LVEF was associated with a decrease of 0.04 units of maximal NC/C ratio. Incorporating PA (when measured in total MET-min/week) with LVEF as a cross-product significantly modified this relationship by augmenting the negative relationship between LVEF and NC/C ratio (p=0.03).

### Association between covariates and NC/C ratio in the fully adjusted models

Tables 3 and 4 show the results of the fully adjusted multivariate regression models carried out between both methods of PA measurement and maximal NC/C ratio in our ‘dose–response’ analysis. Both models showed positive associations between female sex and LVEDV and maximal NC/C ratio, as well as negative associations between LV mass and LVEF and maximal NC/C ratio. The model investigating PA in total MET-min/week showed a positive association between age and NC/C ratio. The model investigating PA in overall 7-day average acceleration showed a negative association between BMI and maximal NC/C ratio.

**Table 3:** Fully adjusted multivariate regression model from analysis of the relationship between PA in total MET-min/week and maximal NC/C ratio (p values less than 0.05 are in bold)

Covariate	Effect estimate	SE	95% CI		P value
			Low	High	
Age (per SD: 7.68 years)	0.041	0.015	0.000	0.077	**0.031**
Sex (male)	−0.169	0.050	−0.267	−0.071	**0.001**
Ethnicity (Caucasian)	0.210	0.182	−0.148	0.568	0.249
Height (per SD: 9.26 cm)	−0.009	0.028	−0.065	0.037	0.610
BMI (per SD: 4.19 kg/m^2^)	−0.034	0.017	−0.071	0.000	0.054
SBP (per SD: 18.0 mm Hg)	−0.018	0.018	−0.072	0.018	0.344
DBP (per SD: 9.64 mm Hg)	−0.010	0.019	−0.058	0.029	0.640
HR (per SD: 11.7 bpm)	0.012	0.012	−0.023	0.047	0.476
Average household income before tax:					
Less than £18 000	0.014	0.053	−0.089	0.117	0.788
£31 000–£51 999	−0.053	0.041	−0.134	0.027	0.192
£52 000–£100 000	−0.067	0.049	−0.163	0.029	0.170
Greater than £100 000	−0.005	0.073	−0.147	0.138	0.946
Degree level or professional education	−0.041	0.032	−0.105	0.022	0.204
Townsend deprivation index (per SD: 2.68)	−0.021	0.016	−0.054	0.011	0.178
Smoking status:					
Never smoked	−0.028	0.078	−0.181	0.125	0.718
Previous smoker	−0.052	0.079	−0.208	0.103	0.509
Regular alcohol use	−0.021	0.032	−0.083	0.042	0.519
Diabetes mellitus	−0.082	0.070	−0.220	0.055	0.240
Cardiovascular disease	−0.014	0.058	−0.127	0.100	0.811
Hypertension	−0.084	0.057	−0.196	0.027	0.138
Antihypertensive use	0.017	0.064	−0.108	0.142	0.790
Statin use	0.002	0.044	−0.084	0.089	0.956
LVEDV (per SD: 33.3 mL/m^2^)	0.166	0.025	0.112	0.210	**<0.001**
LV mass (per SD: 24.1 g/m^2^)	−0.120	0.028	−0.174	−0.065	**<0.001**
LVEF (per SD: 5.91%)	−0.033	0.016	−0.064	−0.016	**0.039**
Total MET-min/week (per IQR: 3505 MET-min/week)	−0.001	0.019	−0.039	0.037	0.966

BMI, body mass index; DBP, diastolic blood pressure; LV, left ventricular; LVEDV, LV end-diastolic volume; LVEF, LV ejection fraction; MET, metabolic equivalent of task; NC/C, non-compaction/compaction; PA, physical activity; SBP, systolic blood pressure.

**Table 4:** Fully adjusted multivariate regression model from analysis of the relationship between PA in average acceleration and maximal NC/C ratio (p values less than 0.05 are in bold)

Covariate	Effect estimate	SE	95% CI		P value
			Low	High	
Age (per SD: 7.68 years)	0.030	0.023	0.000	0.068	0.077
Sex (male)	−0.175	0.050	−0.273	−0.077	**<0.001**
Ethnicity (Caucasian)	0.167	0.184	−0.194	0.528	0.365
Height (per SD: 9.26 cm)	−0.019	0.028	−0.065	0.028	0.416
BMI (per SD: 4.19 kg/m^2^)	−0.046	0.021	−0.088	−0.008	**0.014**
SBP (per SD: 18.0 mm Hg)	−0.018	0.018	−0.072	0.018	0.366
DBP (per SD: 9.64 mm Hg)	−0.010	0.019	−0.058	0.029	0.622
HR (per SD: 11.7 bpm)	0.012	0.012	−0.023	0.047	0.638
Average household income before tax:					
Less than £18 000	0.017	0.053	−0.088	0.122	0.751
£31 000–£51 999	−0.052	0.041	−0.132	0.029	0.210
£52 000–£100 000	−0.069	0.049	−0.164	0.027	0.158
Greater than £100 000	−0.014	0.073	−0.157	0.129	0.845
Degree level or professional education	−0.036	0.032	−0.100	0.027	0.263
Townsend deprivation index (per SD: 2.68)	−0.021	0.016	−0.054	0.011	0.174
Smoking status:					
Never smoked	−0.019	0.078	−0.172	0.134	0.807
Previous smoker	−0.042	0.080	−0.198	0.114	0.600
Regular alcohol use	−0.020	0.032	−0.082	0.043	0.536
Diabetes mellitus	−0.083	0.070	−0.220	0.054	0.235
Cardiovascular disease	−0.022	0.058	−0.136	0.091	0.698
Hypertension	−0.091	0.057	−0.202	0.021	0.110
Antihypertensive use	0.019	0.063	−0.105	0.144	0.761
Statin use	0.002	0.044	−0.084	0.088	0.962
LVEDV (per SD: 33.3 mL/m^2^)	0.165	0.025	0.116	0.214	**<0.001**
LV mass (per SD: 24.1 g/m^2^)	−0.110	0.028	−0.165	−0.055	**<0.001**
LVEF (per SD: 5.91%)	−0.034	0.016	−0.065	−0.003	**0.034**
Seven-day average acceleration (per IQR: 13.4 milligravity)	−0.047	0.031	−0.110	0.015	0.133

BMI, body mass index; DBP, diastolic blood pressure; LV, left ventricular; LVEDV, LV end-diastolic volume; LVEF, LV ejection fraction; MET, metabolic equivalent of task; NC/C, non-compaction/compaction; PA, physical activity; SBP, systolic blood pressure.

### Relationship between PA and clinical cardiac parameters

Across all levels of adjustment, there was no significant relationship between PA and LVEF with both methods of PA measurement. However, with full adjustment, there was a significant positive relationship between PA (in both measurement methods) and LV mass, with a similar significant relationship also observed at limited adjustment when PA was measured in 7-day average acceleration. The analysis of these relationships is shown in table 5. In addition, there was a significant positive relationship between PA (in both measurement methods) and LVEDV across all adjustments (full adjustment in total MET-min/week: ß=1.509 per 1 IQR increment, 95% CI 0.028 to 2.990, p=0.046; full adjustment in 7-day average acceleration: ß=2.474 per 1 IQR increment, 95% CI 0.053 to 4.895, p=0.045).

**Table 5:** Associations between PA (in both methods of measurement) and LVEF, and PA and LV mass

Clinical cardiac parameter	PA measurement method	Model								
		Unadjusted			Limited adjustment			Full adjustment		
		Effect estimate	95% CI	P value	Effect estimate	95% CI	P value	Effect estimate	95% CI	P value
LVEF	Total MET-min/week	−0.440	−0.886 to 0.006	0.053	−0.415	−0.863 to 0.032	0.069	−0.233	−0.687 to 0.221	0.312
	Seven-day average acceleration	−0.501	−1.044 to 0.041	0.070	−0.514	−1.079 to 0.050	0.073	−0.231	−0.895 to 0.433	0.493
LV mass	Total MET-min/week	1.388	−0.222 to 2.998	0.091	0.969	−0.178 to 2.115	0.097	1.344	0.380 to 2.307	0.006
	Seven-day average acceleration	2.329	−0.153 to 4.811	0.066	2.587	−0.767 to 4.407	0.005	3.480	1.839 to 5.121	<0.001

Effect estimate reflects an LVEF (in %) or LV mass (in g/m^2^) change per IQR of either total MET-min/week or 7-day average acceleration.

Effect estimate reflects an LVEF (in %) or LV mass (in g/m^2^) change per IQR of either total MET-min/week or 7- day average acceleration.

LV, left ventricular; LVEF, LV ejection fraction; MET, metabolic equivalent of task; PA, physical activity.

### Dose response and categorical intensity analysis between PA and maximal NC/C ratio

Table 6 shows the pooled multivariate regression analysis, after multiple imputation, between PA in MET-min/week at walking, moderate and vigorous PA intensities and maximal NC/C ratio, as well as between PA at categorical low versus moderate, and categorical low vsersu high PA intensity, and maximal NC/C ratio. At all levels of adjustment, no significant relationship was found.

**Table 6:** Association between PA (measured in [1] walking, moderate and vigorous MET-min/week and [2] low vs moderate and high PA intensity) and maximal NC/C ratio

PA measurement method	Model								
	Unadjusted			Limited adjustment			Full adjustment		
	Effect estimate	95% CI	P value	Effect estimate	95% CI	P value	Effect estimate	95% CI	P value
Total walking MET-min/week	0.006	−0.024 to 0.037	0.68	0.005	−0.025 to 0.035	0.76	−0.007	−0.037 to 0.023	0.65
Total moderate MET-min/week	0.001	−0.033 to 0.035	0.95	0.002	−0.031 to 0.036	0.88	−0.018	−0.052 to 0.016	0.31
Total vigorous MET-min/week	0.016	−0.007 to 0.039	0.19	0.021	−0.001 to 0.044	0.06	0.006	−0.017 to 0.029	0.60
Low versus moderate categorical activity	0.045	−0.039 to 0.130	0.29	0.048	−0.035 to 0.131	0.26	0.022	−0.060 to 0.104	0.60
Low versus high categorical activity	0.009	−0.073 to 0.092	0.83	0.022	−0.058 to 0.104	0.58	−0.037	−0.119 to 0.046	0.39

Effect estimate reflects an NC/C ratio change per IQR MET-min/week.

MET, metabolic equivalent of task; NC/C, non-compaction/compaction; PA, physical activity.

## Discussion

In our study, the first to examine the relationship between PA and LV trabeculation extent in a community-based cohort using CMR imaging, the following observations were made. First, there was no linear relationship between PA and maximal NC/C ratio for both PA measurement methods. Second, there was no significant difference in maximal NC/C ratio between the lowest and highest PA extreme groups for both PA measurement methods. Third, PA (measured in total MET-min/week) augmented the negative relationship between LVEF and maximal NC/C ratio. Finally, age and LVEDV exhibited a significantly positive linear relationship with maximal NC/C ratio, while male sex, BMI, LV mass and LVEF exhibited a significantly negative linear relationship with maximal NC/C ratio.

Studies that previously hypothesised that LV trabeculation extent may participate in the exercise-induced cardiac remodelling process using models of pregnancy and extreme athletically trained PA levels found increased trabeculation extent in tandem with other phenotypical LV changes expected under these preload increasing conditions, such as increased LV mass, LVEDV and LV cavity size.^5 6^ No linear relationship was identified between PA and LV trabeculation extent, despite evidence to suggest exercise induced cardiac remodelling, namely a significant positive linear relationship between PA and LVEDV, and PA and LV mass. This suggests that within a community-based population, trabeculation extent is not a cardiac phenotype that is significantly sensitive enough to be influenced by PA, reinforced by the lack of a significant ‘dose–response’ relationship found between PA in MET-min/week and maximal NC/C ratio at vigorous PA intensity levels. In addition, there was no significant effect of extreme PA on trabeculation extent in our extreme groups analysis. This finding contrasts with the results of Gati’s study in 2013 of a similar sample size, where athletes displayed a higher prevalence of increased LV trabeculation (18.3%) compared with controls (7.0%).^6^

It could therefore be suggested that there exists a PA threshold that must be exceeded for increased trabeculation extent to manifest as a phenotypical change in response to increased PA. As Gati’s study selected athletes ‘competing at regional or national levels’ that were aged between 14 years and 35 years, it was more likely to select for elite athletes compared with that of our cohort, with an older age range and more reflective of a general community-based population. It is therefore possible that the PA levels reached by the cohort in our study were not high enough to produce trabeculation changes of sufficient magnitude to be detected despite our large sample size.

Effect size modification by covariates was also investigated. In the fully adjusted model, including LVEF as a cross-product produced a significant effect size modification in the relationship between PA (in total MET-min/week) and maximal NC/C ratio. Given the significant negative relationship found in our study between LVEF and maximal NC/C ratio, the effect modification analysis suggests that PA may have some degree of further negative influence on this relationship.

Our study also echoed previous research within another community-based cohort—the Multi-Ethnic Study of Atherosclerosis (MESA), where higher PA levels resulted in both an increased LVEDV and a decreased resting HR.^16^ Further research performed using the MESA cohort also identified influencing factors on trabeculation extent similar to our study, where both female sex and higher LVEDV were associated with a higher maximal NC/C ratio. However, this study identified no association between LVEF and maximal NC/C ratio, which contrasts with our study’s findings.^17^

Evidence from another recent study has further validated our finding of an association between LVEDV and trabeculation extent. This was undertaken in a healthy Singaporean Chinese cohort, which found LVEDV to also be positively concordant with LV trabeculation extent, measured by fractal dimension (FD) analysis.^18^ This study also reported a positive association between LV mass and LV trabeculation extent whereas, in contrast, our study found a negative association between LV mass and maximal NC/C ratio. This can however be explained by hearts with higher LV mass exhibiting a thicker compacted myocardium, thereby reducing maximal NC/C ratio values in our study by augmenting the denominator.

### Limitations

There were some limitations of the data relevant to our study. The total MET-min/week measurement of PA was self-reported, reducing the consistency of recordings due to differing interpretations of the questionnaire by each participant. The calculation process in the questionnaire also did not take into account more precise measures of the amount of each exact type of PA undertaken, instead grouping activities into three relative MET intensities for calculation. Seven-day average acceleration was a more objective alternate measurement gained from the wrist-worn accelerometer, but the 1 week sample period may not have produced average estimates as accurate as those from a longer sample period. Also, the values produced by average measurements may not have been large enough to reflect a potential ‘threshold dose’ of PA, above which an effect on maximal NC/C ratio may have been observed.

Our study was also limited in the trabeculation measurement method used. As the maximal NC/C ratio was taken for the LV globally, regional distribution of LV trabeculation was therefore not considered, meaning that more detailed region-specific analysis could not be performed, such as the impact of PA purely on apical trabeculation extent. Also, much previous analysis of LV trabeculation has been performed using echocardiographic data,^5 6^ which is the most common method of assessing LV trabeculation extent in the context of clinically diagnosing LVNC.^19^ Such data are unavailable from the UK Biobank, which limit potential parallel analyses comparing echocardiographic and CMR data for each participant. The accuracy of estimates generated using multiple imputation to account for missing data is most optimal if the missing values are ‘missing at random’, hence depend on observed existing data rather than unobserved external factors. Additionally, as our study was cross-sectional, relevant time periods for physiological cardiac remodelling were not defined; therefore, concrete inferences about whether a causal link exists between PA levels and trabeculation extent should be interpreted with caution in the absence of longitudinal data.

### Future direction

While our study used well-known measures of both trabeculation and PA, it would be valuable to additionally explore this relationship using different approaches to these measurements.

Increasing the time over which the average acceleration is measured would increase its validity by accounting further for the variation that exists between individuals’ activity patterns. In addition, using the accelerometer to gain a measurement of direct activity intensity may better differentiate those that indeed undergo the most athletic activity in the cohort, which would introduce a more robust analysis of the relationship at low and high PA extremes.

Trabeculation extent could alternatively be measured by FD. This is a more automated measurement than the NC/C ratio, which is based on the fractal biology in which trabeculation is structured to measure the complexity of the trabeculation in short-axis CMR slices. While removing the possibility of analysis of compacted myocardial thickness (due to no involvement of compacted wall measurement), FD takes trabeculation measurement into account across the whole LV, allowing for region-specific analysis as well as demonstrating marginally higher intraobserver reproducibility than NC/C ratio measurements.^20^

## Conclusions

In the first study to investigate the relationship between PA and LV trabeculation extent within a community-based sample population using CMR imaging, our results showed no significant relationship between PA and maximal NC/C ratio in a cohort that demonstrated characteristics of exercise-induced cardiac remodelling. At the levels of activity recorded, there was no evidence to suggest that trabeculation changes occur as an epiphenomenon to other processes in this remodelling. The possibility of whether exercise-related changes in trabeculation extent occur above a certain threshold of PA, and where this threshold lies, remains to be investigated.

**Key messages**

**What is already known on this subject?**

- It has been shown that postnatal changes that affect cardiac loading conditions affect left ventricular (LV) trabeculation extent, such as in pregnancy and during extreme athletic-level physical activity (PA). Excessive levels of LV trabeculation form a diagnostic phenotype in left ventricular non-compaction cardiomyopathy.

**What might this study add?**

- This study provides insight into the relationship between PA and LV trabeculation extent, at levels of PA typical of a non-athletic community-based middle-aged population cohort analysed using cardiovascular magnetic resonance (CMR) imaging. We observed no relationship at or between extremes of PA within our cohort of 1030 individuals, despite evidence to suggest exercise-induced cardiac remodelling in other parameters such as left ventricular end-diastolic volume and LV mass.

**How might this impact on clinical practice?**

- At the PA levels typical of a community-based population, there is no evidence to suggest that excessive trabeculation, if observed, occurs as an epiphenomenon to other exercise induced cardiac remodelling processes.
