# Supplementary material for: Physical activity and left ventricular trabeculation in the UK Biobank community-based cohort study
Source: Heart. 2019 Feb 5;105(13):990–8. doi: 10.1136/heartjnl-2018-314155 (PMC6582810; doi:10.1136/heartjnl-2018-314155)
Supplement: Supplementary data [file heartjnl-2018-314155supp001.pdf]

Supplementary Material

Table 1 – Example MET values assigned to various forms of PA, grouped into light, moderate, and vigorous PA levels (11). The MET is a physiological measure of energy expenditure assigned to a particular PA. Since one MET is defined as the energy spent while an individual is at rest, MET is a measure of the intensity of the activity compared to rest. For instance, a MET value of 7 applied to the activity would mean that the activity expends 7 times the amount of energy (typically the amount of calories) compared to rest.

**Light activity (<3.0 METs)**	**Moderate Activity (3.0 – 6.0 METs)**	**Vigorous Activity (>6.0 METs)**
Walking – slowly = 2.0	Walking – very brisk = 5.0	Walking/hiking (4-5mph) = 7.0<br>Jogging at 6mph = 10.0
Sitting – using computer = 1.5	Cleaning – heavy = 3.0-3.5<br>(washing windows, vacuuming, mopping)	Shovelling = 7.0-8.5
Standing – light work = 2.0-2.5<br>(cooking, washing dishes)	Mowing lawn = 5.5<br>(walk power mower)	Carrying heavy loads = 7.5
Fishing – sitting = 2.0<br>Playing most instruments = 2.0-2.5	Bicycling – light effort (10-12mph) = 6.0<br>Badminton – recreational = 4.5<br>Tennis – doubles = 5.0	Bicycling fast (14-16mph) = 10.0<br>Basketball game = 8.0<br>Soccer casual = 7.0<br>Tennis – singles = 8.0

(1) In a typical **week**, on how many **days** did you walk for at least 10 minutes at a time? (Includes walking that you do at work, travelling to and from work, and for sport or leisure)
(2) How many **minutes** did you usually spend walking on a typical **day**?
(3) In a typical **week**, on how many **days** did you do 10 minutes or more of moderate physical activities like carrying light loads, cycling at normal pace?
(4) How many **minutes** did you usually spend doing moderate activities on a typical **day**?
(5) In a typical **week**, how many **days** did you do 10 minutes or more of vigorous physical activity? (These are activities that make you sweat or breathe hard such as fast cycling, aerobics, heavy lifting)
(6) How many **minutes** did you usually spend doing vigorous activities on a typical **day**?

Figure 1 - Example questions for participants in the self-reported IPAQ questionnaire.

Walking MET-minutes/week = 3.3 x walking days (1) x walking minutes (2)

Moderate MET-minutes/week = 4.0 x moderate-intensity days (3) x moderate-intensity activity minutes (4)

Vigorous MET-minutes/week = 8.0 x vigorous-intensity days (5) x vigorous-intensity activity minutes (6)

Total MET minutes per week = Walking + Moderate + Vigorous MET-min/week scores

Figure 2 - Calculations culminating in the total MET minutes per week, using information gained from the IPAQ questions in figure 1 (Craig et al., 2003).

Table 2 – Power calculations based on potential sample sizes against a range of R^2 values, with a p value less than 0.05 indicating statistical significance.

		Sample size		
		250	500	1000
R^2	0.05	45.6%	85.9%	99.8%
	0.10	86.5%	99.9%	100%
	0.15	98.6%	99.9%	100%
	0.20	99.9%	100%	100%

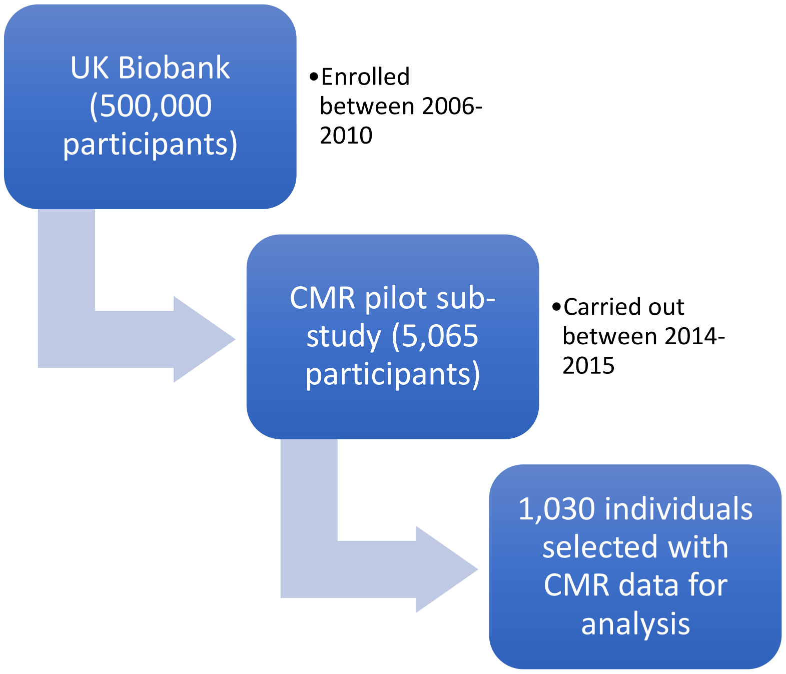

Figure 3 – A flowchart summarising the selection process for the individuals investigated in this study.

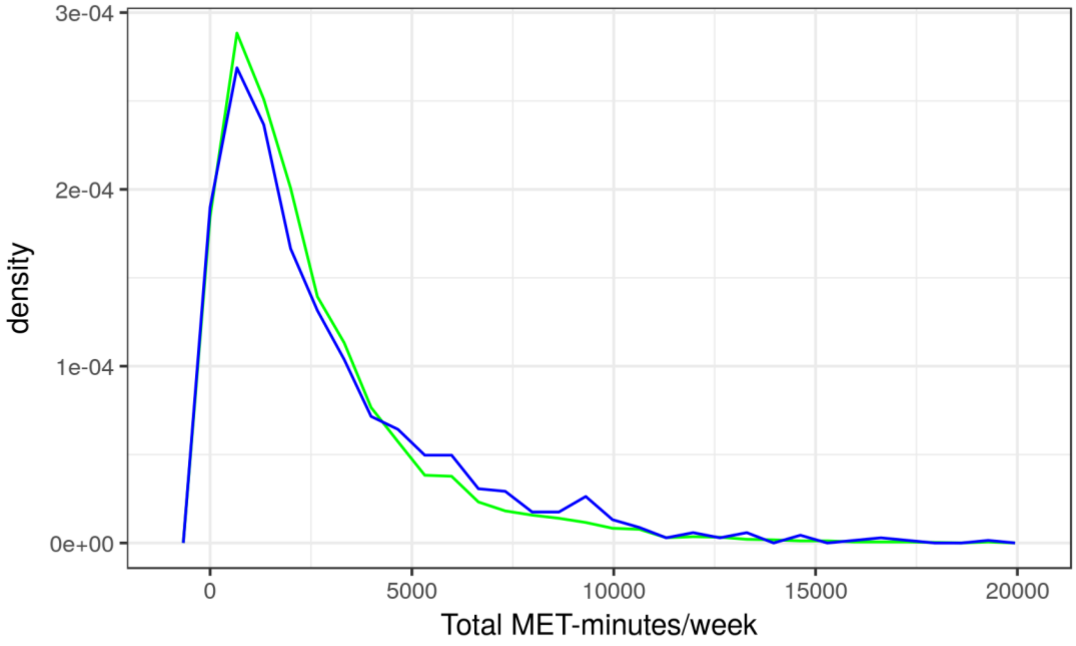

Figure 4 – A density plot demonstrating the distribution of PA in total MET-minutes per week across the study cohort of 1030 individuals (blue) relative to that of the original CMR pilot study of 5065 individuals (green)

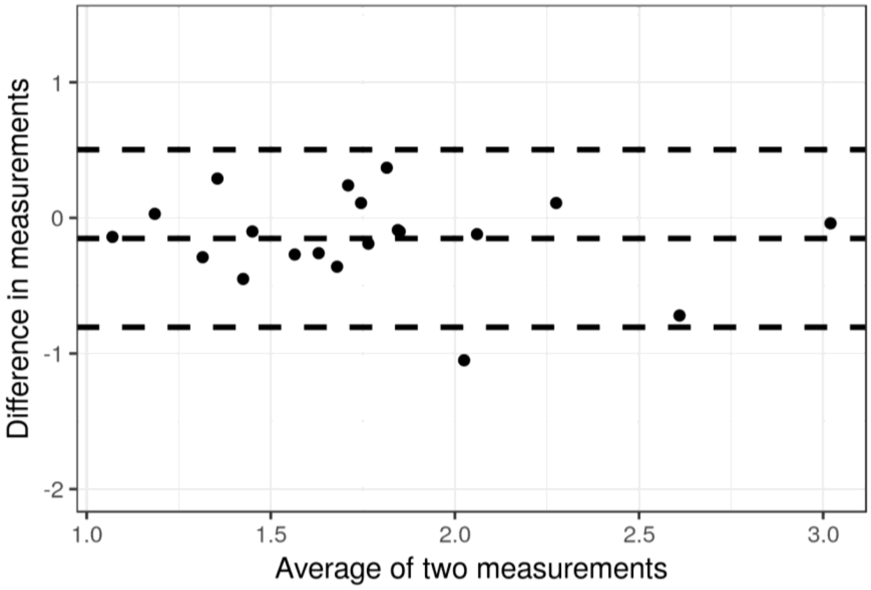

Figure 5 – A Bland-Altman plot demonstrating the inter-observer variability of NC/C ratio values in 20 studies

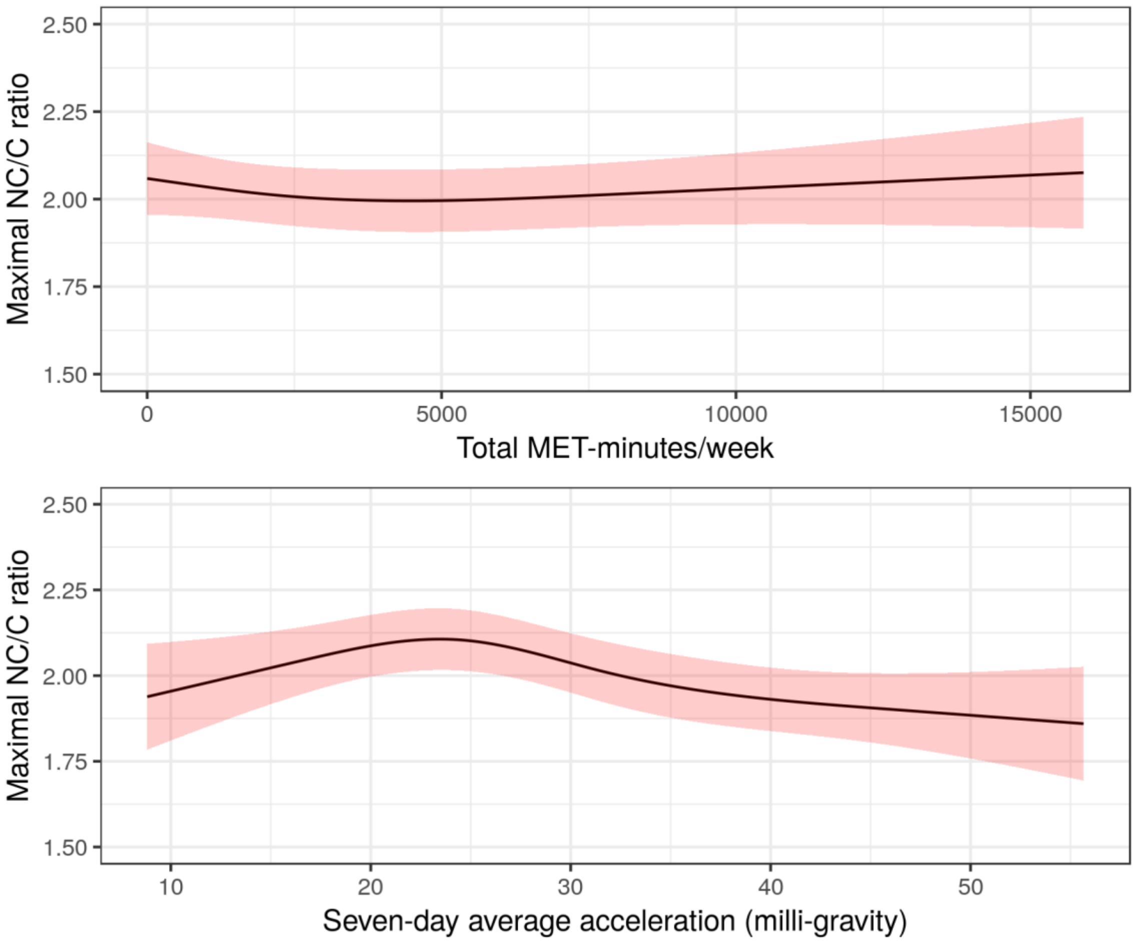

Figure 6 – Association between restricted-cubic-spline-transformed PA measurements and maximal NC/C ratio. The line and the shaded area represent the predicted mean and 95% confidence interval of maximal NC/C ratio. The plots did not demonstrate a clear evidence of non-linear relationship.

Appendix 1:

Methodology of PA intensity category definition

In addition to our other methods of PA measurement, the cohort was split categorically into low, moderate, and high PA intensity levels using criteria as per official IPAQ recommendations and guidance. Moderate PA comprised either a) Over or equal to 3 days of vigorous intensity PA of at least 20 minutes/day OR b) Over or equal to 5 days of moderate-intensity PA and/or walking of at least 30 minutes/day OR c) Over or equal to 5 days of any combination of walking, moderate or vigorous-intensity PA achieving a minimum of at least

600 MET-minutes/week. High PA comprised either a) vigorous intensity PA on at least 3 days achieving a minimum total PA of 1500 MET-minutes/week OR b) Over or equal to 7 days of any combination of walking, moderate or vigorous-intensity PA achieving a minimum total PA of 3000 MET-minutes/week. Low PA was defined in those individuals not meeting criteria for moderate or high PA.
